# Glutathione increase by the n‐butanoyl glutathione derivative (GSH‐C4) inhibits viral replication and induces a predominant Th1 immune profile in old mice infected with influenza virus

**DOI:** 10.1096/fba.2018-00066

**Published:** 2019-03-13

**Authors:** Donatella Amatore, Ignacio Celestino, Serena Brundu, Luca Galluzzi, Paolo Coluccio, Paola Checconi, Mauro Magnani, Anna Teresa Palamara, Alessandra Fraternale, Lucia Nencioni

**Affiliations:** ^1^ Deparment of Public Health and Infectious Diseases Istituto Pasteur Italia‐Fondazione Cenci‐Bolognetti, Sapienza University of Rome Rome Italy; ^2^ Department of Biomolecular Sciences University of Urbino Carlo Bo Urbino (PU) Italy; ^3^ Department of Human Sciences and Promotion of the Quality of Life IRCCS San Raffaele Pisana, San Raffaele Roma Open University Rome Italy

**Keywords:** aging, oxidative stress, redox state, Th response, viral infection

## Abstract

During aging, glutathione (GSH) content declines and the immune system undergoes a deficiency in the induction of Th1 response. Reduced secretion of Th1 cytokines, which is associated with GSH depletion, could weaken the host defenses against viral infections. We first evaluated the concentration of GSH and cysteine in organs of old mice; then, the effect of the administration of the N‐butanoyl GSH derivative (GSH‐C4) on the response of aged mice infected with influenza A PR8/H1N1 virus was studied through the determination of GSH concentration in organs, lung viral titer, IgA and IgG1/IgG2a production, and Th1/Th2 cytokine profile. Old mice had lower GSH than young mice in organs. Also the gene expression of endoplasmic reticulum (ER) stress markers involved in GSH metabolism and folding of proteins, that is, Nrf2 and PDI, was reduced. Following infection, GSH content remained low and neither infection nor GSH‐C4 treatment affected Nrf2 expression. In contrast, PDI expression was upregulated during infection and appeared counterbalanced by GSH‐C4. Moreover, the treatment with GSH‐C4 increased GSH content in organs, reduced viral replication and induced a predominant Th1 response. In conclusion, GSH‐C4 treatment could be used in the elderly to contrast influenza virus infection by inducing immune response, in particular the Th1 profile.

**Abbreviations**
ER: endoplasmic reticulum
GSH: reduced glutathione
GSH‐C4: N‐butanoyl GSH derivative
IgG: immunoglobulin G
IL: interleukin
Nrf2: nuclear factor erythroid2‐related factor 2
PDI: protein disulfide isomerase
Th: T helper

## 1 INTRODUCTION

Influenza represents a global concern and a challenge for public health systems because of high rates of hospitalization and deaths every year. The infection leads to self‐limiting upper respiratory tract disease that resolves in few days, but unfortunately some individuals develop a fatal infection. Several factors influence the outcome of the infection among which the interplay between virus and host cell response plays a major role. The risk of influenza‐associated death is higher among elderly (persons aged >65 years).^1^

The aged immune system is characterized by an imbalance in the Th1/Th2 response and the lack of an efficient Th1 response puts the elderly at risk to exacerbation of the disease state.^1^

The aging process is accompanied by complex age‐related changes that affect the cell redox homeostasis, the immunity and the host defenses against viral infections. In particular, old individuals are characterized by chronic oxidative stress and low‐grade inflammation, referred to as “inflamm‐aging,” responsible for a blunted immune response, thus increasing the susceptibility of this population to infections and predisposing them to a reduced response to vaccination.^2^ Age‐related decline in the intracellular content of the tripeptide glutathione (GSH) has been observed in various cells and tissues.^3^ GSH depletion impairs the ability of macrophages to process antigens and secrete interleukin (IL)‐12 leading to the polarization toward Th2 response patterns; on the contrary, high GSH content favors the Th1 response.^4^ IL‐12 is produced by macrophages infiltrating the influenza virus infected‐epithelium as the first response cytokine, and its production at the site of infection may direct an early immune defense to alleviate the severity of infection.^5^ Unfortunately, several macrophage functions are compromised with age, including cytokine and chemokine production.^6^ Reduced age‐associated IL‐12 production may be partly attributable to the decrease in GSH content within the aging macrophage.^6^ Therefore, restoring of GSH content in macrophages may be useful for enhancing the immune response and stimulating Th1 immunity during aging.^7^ Fraternale et al have reported that the N‐butanoyl GSH derivative (GSH‐C4), a molecule able to enter cells more easily than GSH, can shift the immune response toward Th1 type in different immunization models by modulating IL‐12 secretion.^8^, ^9^

Intracellular redox state plays also a pivotal role in regulating the life‐cycle of influenza virus and a depletion of GSH was observed in infected cells.^10^, ^11^, ^12^, ^13^ Counteracting virus‐induced oxidant conditions of infected cells by GSH‐C4 significantly inhibits influenza virus replication both in vitro and in vivo. In particular, GSH‐C4 interferes with the maturation process of viral hemagglutinin (HA) by increasing the reduced form of host‐cell protein disulfide isomerase (PDI).^12^

Therefore, although the antiviral activity of GSH‐C4 against influenza has been demonstrated on young mice,^12^ to date no information is available about its potential role as antiviral and/or immunomodulatory agent in aged mice infected with influenza virus. On these bases, the main aim of this study was to investigate whether GSH‐C4 treatment of influenza A virus infected aged mice was able to affect viral replication and/or induce antiviral Th1 type immune response.

## 2 MATERIALS AND METHODS

In accordance with national law, the experiments described in this manuscript were approved by the Italian Ministry of Health. BALB/c mice (15‐month‐old and 8‐week‐old, Harlan Laboratories) were housed and studied under Institutional Animal Care and Use Committee‐approved protocols.

All animals received humane treatment, and every effort was made to minimize their suffering. Unless otherwise stated, all commercial products cited were used in accordance with the manufacturers’ instructions.

### 2.1 Infection and GSH‐C4 treatment of mice

Fifteen‐month‐old mice that had been lightly anesthetized by isofluorane inhalation were intranasally inoculated with 0.5 Plaque Forming Unit (PFU)/mouse of a mouse‐adapted strain of influenza A/Puerto Rico/8/34 (H1N1; PR8) (infected animals) diluted in 50 µL of sterile phosphate buffered saline (PBS). The day of infection, mice were randomly assigned to treatment groups: a group was intraperitoneally (ip) treated with GSH‐C4 (7.4 mg in 100 µL of 0.9% NaCl, ~370 mg/Kg) twice to each animal, 1 hour before and immediately after infection. Then, mice were treated with GSH‐C4 once a day for the next 7 days (I + GSH‐C4). The other group was ip treated with 100 µL of 0.9% NaCl (I + Placebo). As control of infection, two groups were inoculated with 50 µL of PBS (mock‐infected mice) or treated with GSH‐C4 as described above. Each animal was weighed and rectal temperature was measured. At the end of the experiments, the mice were euthanized with an overdose of tiletamine/zolazepam (800 mg/kg body weight, bw) and xylazine (100 mg/kg bw).

### 2.2 Determination of total IgA content and cytokine production in Bronchoalveolar lavage fluid (BALF)

At day 8 post infection (pi), a sterile 23‐gauge catheter was inserted into the exposed tracheal lumen of euthanized mice. Two instillations of sterile PBS (0.8 mL) containing protease inhibitors (Sigma‐Aldrich, Milan, Italy) were injected through the catheter and aspirated as described elsewhere.^13^ The BALF samples were centrifuged at 1000× *g* for 15 minutes at +4°C and the supernatant stored at −80°C prior to analysis.

Total IgA antibodies, Th1 (IL‐2, IL‐12, IFN‐γ) and Th2 cytokines (IL‐4, IL‐5, IL‐10) concentrations were determined in BALF sample obtained from mock‐infected or infected mice, by a multiplex assay. Plates were read on a Bio‐Plex MAGPIX instrument (Bio‐Rad Laboratories, Inc, Hercules, CA). The IgA concentrations (expressed as nanograms per milliliter) and interleukin concentrations (expressed as picograms per milliliter) were calculated by the use of a standard curve and software provided by the manufacturer (Bio‐Plex manager software, v.6.1). All standards and samples were run in duplicate.

### 2.3 IgG1 and IgG2a determination in plasma

At day 8 pi, blood was withdrawn from retro‐orbital cavity of mock‐infected or infected mice and IgG subtypes were determined using an ELISA technique. Polystyrene microtiter 96‐well plates (Nunc‐Immuno™ MicroWell™ plates, Sigma Aldrich, United States) were coated with a solution of 2.5 µg/mL goat anti‐mouse IgG (IgG1 or IgG2a) (Bio‐Rad, Richmond, CA) in 0.135 mol/L NaCl pH 7.2 and incubated at 37°C overnight. The plates were washed four times with 0.1% Tween 20 in 10 mmol/L NaH_2_PO_4_, 154 mmol/L NaCl, pH 7.0 (TPBS) and blocked with 5% bovine serum albumin (BSA) in PBS for 1 hour at 37°C. After four washings in TPBS, serial dilutions of murine plasma in 50 mmol/L sodium borate, pH 8.5, were added and incubated for 1 hour at 37°C. After four washings in TPBS, 100 µL of goat anti‐mouse IgG‐horseradish peroxidase (HRP) conjugate (Bio‐Rad), diluted 1:1000 in PBS, were added. After incubation for 1 hour at 37°C, serum IgG subtypes were determined using a color development solution containing 2.2 mmol/L *o*‐phenylenediamine. Absorbance was measured at 492 nm on a Model 2550 enzyme immunoassay (EIA) reader.

### 2.4 Assay of viral titers

A portion of lung was removed, weighed, frozen, and stored at −80°C. Total RNA was extracted from thawed lungs that had been homogenized in TRI Reagent (Sigma‐Aldrich, Milan, Italy) (1 mL/75 mg of tissue) with a Polytron homogenizer. The RNA pellet was washed with 1 mL of 75% ethanol (7500× *g* for 5 minutes at +4°C) and air‐dried for 30 minutes. Diethylpyrocarbonate water (100 μL) was added, and tube was heated to 55°C for 15 minutes to facilitate dissolution. The isolated RNA was treated with DNase I (Invitrogen, Life Technologies, Monza, Italy), and its quality and quantity were verified spectrophometrically (Pearl Nanophotometer, IMPLEN, Munich, Germany). The number of viral M1 RNA copies was determined by quantitative real time RT‐PCR using the One Step Influenza A/B r‐gene and Quanti FluA kits (BioMérieux, Florence, Italy).

### 2.5 Quantification of GSH and cysteine in organs

GSH and cysteine in the spleen, lymph nodes, lungs, pancreas, and brain of BALB/c mice were quantified following a HPLC method validated according to US and European standards^14^ and applied for determination of the thiol species in several mouse organs.^15^

### 2.6 NRF2 and PDI expression profile in lungs

Total RNA was isolated as described above according to the manufacturer's instructions. The cDNA was synthesized using the RT^2^ first strand cDNA synthesis kit (Qiagen) from 0.8 µg total RNA (n = 3 per each condition). The qPCR was performed using the primer pairs listed in Table 1. The reactions were performed in duplicate or triplicate in a final volume of 20 µL, using RT^2^ SYBR® Green ROX FAST Mastermix (Qiagen), 200 nmol/L primers, in a RotorGene 6000 instrument (Corbett life science, Sydney, Australia). The amplification conditions were: 95°C for 10 minutes, 40 cycles at 95°C for 10 seconds and 60°C for 50 seconds. A duplicate non‐template control was included in each PCR run. At the end of each run, a melting curve analysis from 60°C to 95°C was performed to ensure the absence of primer dimers or non‐specific products. Relative mRNA expression analysis was calculated using the web‐based RT^2^ Profiler PCR Array data analysis software version 3.5 (SABiosciences), using GAPDH as a reference gene and mock‐infected mice as calibrator.

**Table 1:** Primer pairs used for NRF2 and PDI gene expression

Gene name	Accession number	Primer F (5′‐3′)	Primer R (5′‐3′)
Nfe2l2 (NRF2)	NM_010902	CACATTCCCAAACAAGATGCCT	TATCCAGGGCAAGCGACTCA
P4hb (PDI)	NM_011032	GATCAAGCCCCACCTGATGA	ACCTCTTCAAAGTTCGCCCC
Gapdh	NM_001289726	TGCCCCCATGTTTGTGATG	TGTGGTCATGAGCCCTTCC

### 2.7 Statistical analyses

Statistical significance was evaluated using GraphPad Prism™ software version 6.0. Test details of each experiment are described in the figure legends. *P* values <0.05 were considered significant.

## 3 RESULTS

### 3.1 Old mice contain low quantity of GSH and cysteine in different organs except for the brain

**Figure 1:**
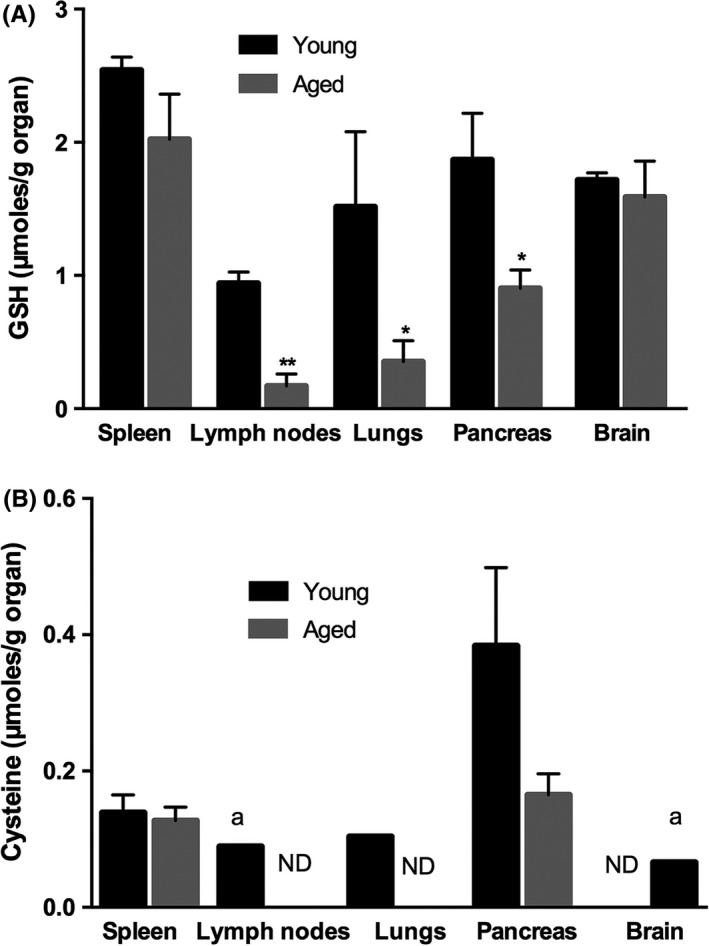
Reduced glutathione (GSH) and cysteine content in organs of young and aged mice. GSH (A) and cysteine (B) contents were quantified in the spleen, lymph nodes, lungs, pancreas and brain of young (8‐week‐old) and aged (15‐month‐old) mice through HPLC according to the procedure described in the Materials and Methods section. Values represent the mean ± SD of three mice, each performed in duplicate (n = 6) and are expressed as µmoles/g organ. Unpaired data were analyzed with the Student's *t* test with Welch correction *P < 0.05; **P = 0.005; vs young mice; a: the value is referred to one mouse; ND: not detectable

Aging is characterized by a progressive decline in GSH concentration in humans and rodents, as well as in senescent cells in culture.^3^ Therefore, at first we evaluated the basal concentrations of GSH and cysteine in spleen, lymph nodes, lungs, pancreas, and brain of aged and young mice. As reported in Figure 1, the content of thiol species of aged mice was lower in all organs. Specifically, statistically significant differences in GSH content were recorded in lymph nodes, lungs, and pancreas. Evident reductions, although not significant, were found in the spleen, while no changes were found in the brain (Figure 1A). Quantification of cysteine was more difficult because of the previously reported low content of this aminoacid in BALB/c mice^14^; however, we observed in pancreas of old mice, where the highest concentration of cysteine was measured, a marked decrease in the aminoacid (*P* = 0.06) (Figure 1B).

### 3.2 Treatment with GSH‐C4 inhibits viral yields in the lungs of infected mice

**Figure 2:**
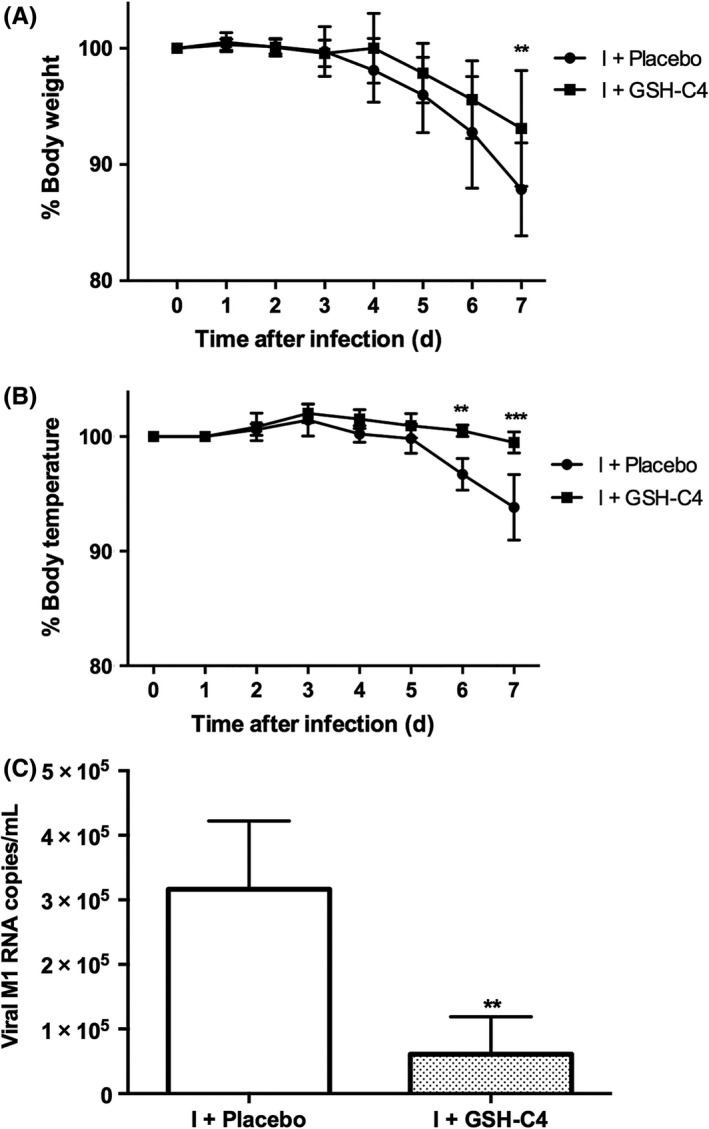
Antiviral effect of GSH‐C4 in aged mice. Fifteen‐month‐old mice were infected with a mouse‐adapted strain of influenza A/Puerto Rico/8/34 (H1N1; PR8) and treated with Placebo (I + Placebo) or GSH‐C4 (I + GSH‐C4) as described in the Materials and Methods section. The graphs represent the percentages of body weight (A) and body temperature (B) vs time 0 recorded in mice during treatment with GSH‐C4 (squares) or Placebo (circles). At day 8 pi, viral M1 RNA copies were measured in lungs (C) by quantitative Real Time (qRT‐PCR) as described in the Materials and Methods section. Results are the mean ± SD of five mice, each run in duplicate (n = 10). (A) and (B): Multiple *t* test followed by Holm‐Sidak method: **P = 0.003; ***P = 0.0004 *vs* I + Placebo; (C): Unpaired data were analyzed with the Student's *t* test with Welch correction: **P = 0.003

We have previously demonstrated that treatment with GSH‐C4 strongly inhibits influenza virus replication in 6‐week‐old mice.^12^ In this paper, the antiviral effect of the GSH derivative in 15‐month‐old mice was evaluated. For all the duration of the treatments, animals were daily monitored for survival, clinical signs of infection, body weight, and body temperature. At selected time, lungs were used for measuring viral titers. According with the results obtained in the young mice,^12^ both groups showed signs of infection starting from day 4 pi, but Placebo‐treated mice were more affected than GSH‐C4‐treated ones. Indeed, loss of body weight (Figure 2A) and decrease in temperature (Figure 2B) were more pronounced in the Placebo‐treated mice, especially late in infection. Furthermore, on day 8 pi, viral M1 RNA copies were significantly reduced in the GSH‐C4‐treated group (Figure 2C).

### 3.3 Treatment with GSH‐C4 increases GSH concentration in organs of infected mice

**Figure 3:**
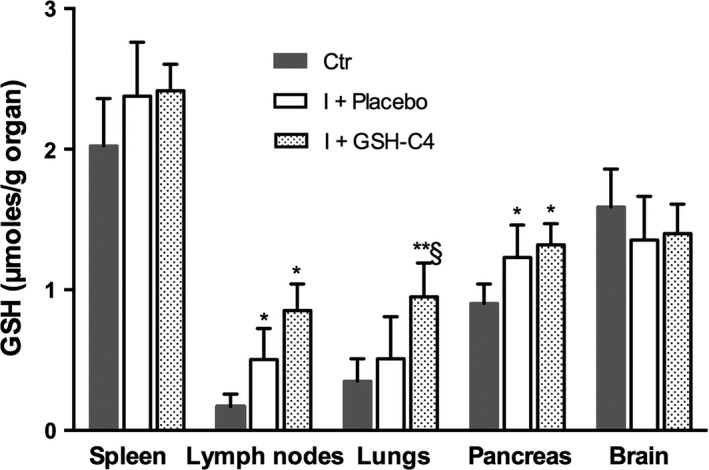
Reduced glutathione (GSH) content in the organs of infected aged mice. Fifteen‐month‐old mice were infected with a mouse‐adapted strain of influenza A/Puerto Rico/8/34 (H1N1; PR8) and treated with Placebo (I + Placebo) or GSH‐C4 (I + GSH‐C4) as described in the Materials and Methods section. At day 8 pi, GSH level was quantified in the spleen, lymph nodes, pancreas, and brain as described in the Materials and Methods section. Values represent the mean ± SD of three mice each performed in duplicate (n = 6). Unpaired data were analyzed with the Student's *t* test with Welch correction: *P < 0.05, **P = 0.002 vs Ctr (mock‐infected mice); §*P* < 0.05 vs I + Placebo

Influenza virus causes redox changes in infected cells and GSH depletion favors virus replication^10^, ^11^, ^12^, ^13^, ^16^, ^17^; hence, at day 8 pi we investigated whether infection and/or GSH‐C4 treatment modified GSH content in spleen, lymph nodes, lungs, pancreas, and brain of old mice. Surprisingly, GSH content was increased in all the organs of infected mice, except in the brain (Figure 3). In details, statistically significant raises were observed in lymph nodes and pancreas while evident increases, although not statistically relevant, were recorded in lungs and spleen. Interestingly, GSH‐C4 treatment further increased GSH level in the lymph nodes and lungs (*P* = 0.06 and 0.03 vs I + Placebo, respectively) while it had no significant effect on the other organs (Figure 3).

### 3.4 Treatment with GSH‐C4 reduces the expression of PDI but not that of Nrf2 in lungs of infected mice

**Figure 4:**
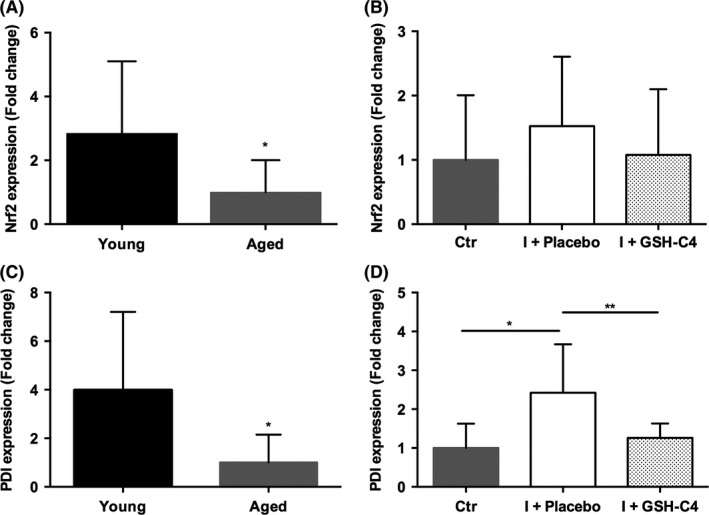
Expression of NRF2 and PDI genes in lung homogenate of young and aged mice and of infected aged mice 15‐month‐old mice were infected with a mouse‐adapted strain of influenza A/Puerto Rico/8/34 (H1N1; PR8) and treated with Placebo (I + Placebo) or GSH‐C4 (I + GSH‐C4) as described in the Materials and Methods section. Lung homogenate was obtained at day 8 pi. The fold change respect to Young (A and C) or to Ctr (mock‐infected mice) (B and D) was calculated from three biological replicates using 2^−ΔΔCt^ method with normalization of the raw data to the reference gene GAPDH. Data analysis was performed using the web‐based RT2 Profiler PCR Array data analysis software (SABiosciences). The error bars represent 95% confidence intervals. Unpaired *t* test with Welch's correction *P < 0.05, **P = 0.0094

Activation of nuclear factor‐erythroid‐2 related factor 2 (Nrf2) may be deregulated during the aging process.^18^ It is also known that Nrf2 controls GSH production and regeneration,^19^ and that a modified expression of Nrf2 can alter influenza virus entry and subsequent replication.^20^ Having found very low quantity of GSH in the lungs of old mice (Figure 1A), we first investigated Nrf2 expression in lung homogenate of old and young mice, then how it was influenced by both infection and GSH‐C4 treatment in old mice. Interestingly, as shown in Figure 4A, Nrf2 expression was significantly lower in old mice respect to the young ones. With regard to infected mice, neither viral infection nor GSH‐C4 treatment significantly affected Nrf2 expression (Figure 4B).

Next, we turned our attention on PDI, a chaperone necessary for proper protein folding, and generally impaired during the aging process.^21^ Previously, PDI has been reported to mediate infection of enveloped viruses^22^ and, in the NCI‐H292 lung cells infected with influenza virus, its expression was increased.^12^ Furthermore, GSH‐C4 treatment, by altering the redox state of both PDI and HA proteins, inhibited viral replication.^12^ Here, we studied the gene expression of PDI in lung homogenate of old and young mice (Figure 4C), and evaluated the effect of infection and GSH‐C4 treatment on its transcription in old mice (Figure 4D). As shown in Figure 4C, the basal expression of PDI was significantly reduced in old mice respect to the young. Accordingly to previous results,^12^ the influenza virus induced a significant up‐regulation of PDI expression, which appeared significantly counterbalanced by GSH‐C4 treatment (Figure 4D).

### 3.5 Treatment with GSH‐C4 induces the production of IgA and IgG in infected mice

**Figure 5:**
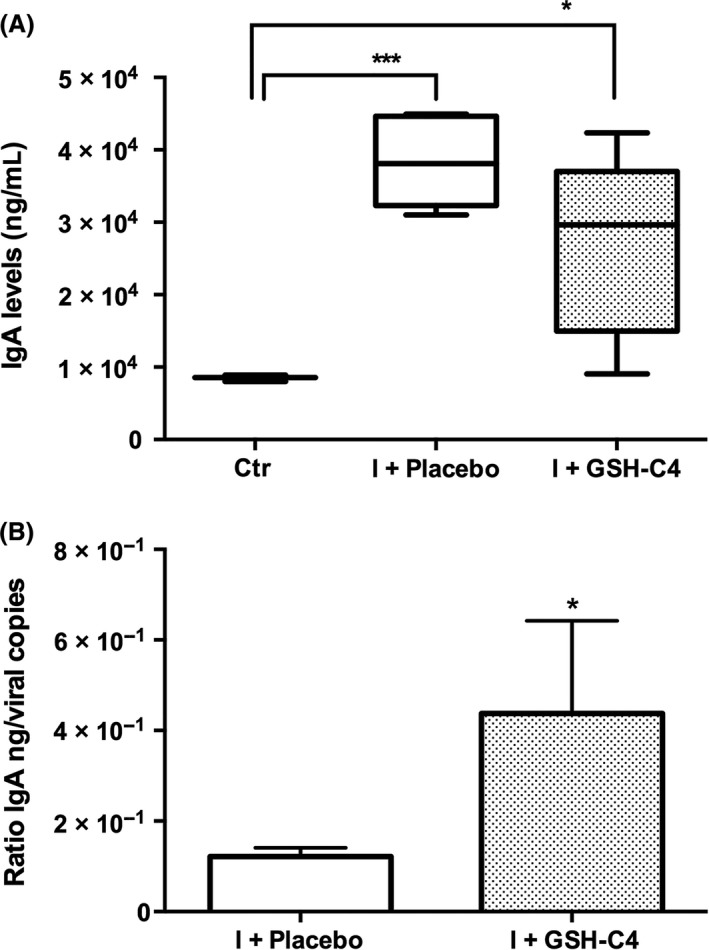
IgA titer in BALF of infected aged mice. (A) IgA content was measured in BALF obtained from Ctr (mock‐infected mice) and infected mice treated with Placebo (I + Placebo) or GSH‐C4 (I + GSH‐C4) at day 8 pi. Mouse infection and the methods used for determination of IgA level are described in the Materials and Methods section. Values represent the mean ± SD of 5 mice, each run in duplicate (n = 10). Unpaired *t* test with Welch's correction: *P < 0.05; ***P = 0.0002 *vs* Ctr. (B) Ratio of IgA ng/viral M1 RNA copies in infected aged mice treated with Placebo or GSH‐C4. Unpaired *t* test with Welch's correction *P = 0.03 vs I + Placebo

The immune system undergoes dramatic age‐associated remodeling, including deficiency in the induction of specific Th1 cells. In order to assess the possible immunomodulatory effect of GSH‐C4 in infected aged mice, IgA titer was measured in BALF collected from mock‐infected (Ctr) and infected mice. As shown in Figure 5, influenza virus caused a significant increase in the IgA concentration (***P = 0.0002). Although the treatment with GSH‐C4 seemed to partially reduce IgA content with respect to Placebo‐infected mice, the ratio IgA titer/viral copies produced in GSH‐C4‐treated mice was significantly higher compared with Placebo‐treated mice (Figure 5B).

**Figure 6:**
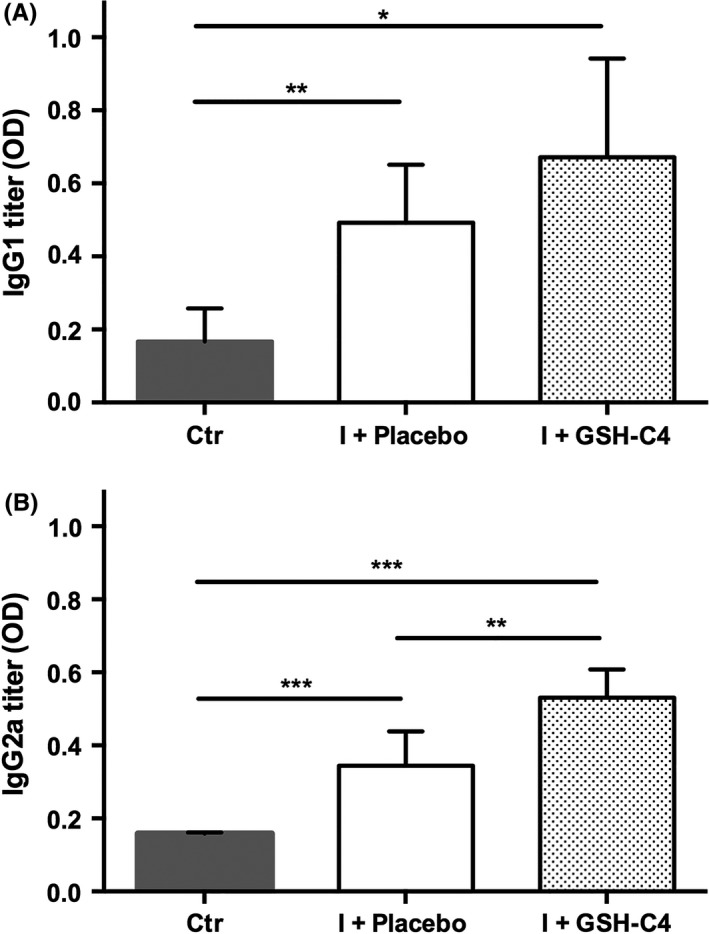
Plasma IgG1 and IgG2a titers in infected aged mice. Fifteen‐month‐old mice were infected with a mouse‐adapted strain of influenza A/Puerto Rico/8/34 (H1N1; PR8) and treated with Placebo (I + Placebo) or GSH‐C4 (I + GSH‐C4) as described in the Materials and Methods section. At day 8 pi, IgG1 (A) and IgG2a (B) were determined by ELISA as described in the Materials and Methods section. Antibody titers are reported as optical density (OD) of the 1/32000 dilution. Values represent the mean ± SD of five mice, each run in duplicate (n = 10). Unpaired data were analyzed with the Student's *t* test with Welch correction: *P = 0.01, **P < 0.005, ***P < 0.0005

In mice, high concentrations of IgG1 are associated with a Th2‐type immune response, while high IgG2a titers are indicative of a Th1 response.^23^ For this reason, the ability of GSH‐C4 to modulate the Th1/Th2 response was investigated by measuring IgG1 and IgG2a titer in plasma of infected mice. We found that viral infection induced both IgG1 and IgG2a production (Figure 6A,B, respectively) and that GSH‐C4 treatment increased both IgG subtypes, although IgG2a expression reached a concentration that was statistically higher than Placebo treatment (Figure 6B).

### 3.6 Treatment with GSH‐C4 induces a predominant Th1 cytokine profile

**Figure 7:**
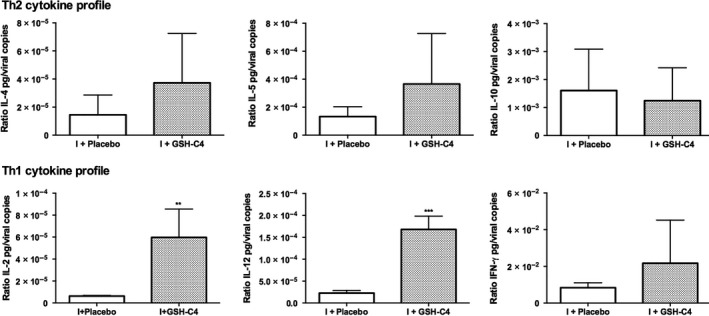
Th1 and Th2 cytokine profile in infected aged mice. Production of Th2 cytokines (IL‐4, IL‐5, IL‐10) (above) and of Th1 cytokines (IL‐2, IL‐12, IFN‐γ) (below) in BALF obtained from infected mice either treated with Placebo (I + Placebo) or GSH‐C4 (I + GSH‐C4) at day 8 pi. Cytokine assays were performed as described in the Materials and Methods section. Values, reported as ratio of IL pg/viral copies, represent the mean ± SD of five mice, each run in duplicate (n = 10). Unpaired data were analyzed with the Student's *t* test with Welch correction: **P = 0.0098, ***P = 0.0003 vs I + Placebo

Finally, to evaluate the capacity of GSH‐C4 to shift the immune response toward Th1 type in old infected mice, the production of Th1 (IL‐2, IL‐12, IFN‐γ) and Th2 (IL‐4, IL‐5, IL‐10) cytokines was investigated. Eight days after infection, quantitative analysis of cytokines performed in BALF of infected mice, revealed that treatment with GSH‐C4 predominantly influenced differentiation toward the Th1 phenotype. In fact, we found that GSH‐C4 treatment enhanced the production of all the cytokines except IL‐10, but only Th1 cytokine production, and in particular IL‐2 and IL‐12, were significantly higher in GSH‐C4‐treated mice compared with those measured in mice receiving Placebo, shifting Th1/Th2 response in favor of Th1 (Figure 7).

## 4 DISCUSSION

In this study, we demonstrate that basal GSH content is lower in several organs of aged compared with young mice. Since human influenza strains replicate almost exclusively in superficial cells of the respiratory tract,^24^ we chose to evaluate most of parameters only in lungs isolated from infected mice. The significant decrease in mRNA expression of Nrf2, the transcription factor that controls the expression of the glutamate cysteine ligase, the rate limiting enzyme of GSH de novo synthesis,^19^ could explain the observed GSH low concentration in the lungs of old mice. Differently from what reported in previous papers, where influenza virus infection was associated with the depletion of GSH,^10^, ^11^, ^12^ in this study an increase in the tripeptide in old infected animals was observed. Indeed, old mice did not possess physiological GSH concentrations at the time of infection (Figure 1A). Thus, we hypothesize that, following infection of old animals, different intracellular pathways can be activated depending on their intracellular redox state. Further studies are in progress to unravel this concern.

Regarding the effect of GSH‐C4, the peritoneal administration of this molecule in old mice increased GSH concentration in lymph nodes and lungs of mice. On the contrary, no GSH raise was measured in the organs where GSH concentration was not depleted, suggesting that it may have different effects on the GSH content and the redox‐mediated regulation of intracellular signaling depending on the redox status of the cell.

Regarding the mechanism of action through which GSH‐C4 could increase GSH content, we hypothesize that the ectoenzyme λ‐glutamyl transpeptidase (λGT) can catalyze the transfer of the λ‐glutamyl moiety from GSH‐C4 onto an acceptor molecule, thereby generating the CysGly conjugate.^25^ CysGly may be hydrolyzed by ectopeptidases to cysteine and glycine, which can be used for cellular GSH synthesis.^19^ Hence, in a GSH‐depleted cell, GSH‐C4 treatment can increase intracellular thiol content in the form of GSH‐C4 and by raising GSH level.

Previously we have demonstrated that influenza virus infection enhances expression of PDI and that GSH‐C4 treatment reduces the enzyme's ability to fold correctly HA by interfering with the oxidation state of PDI.^12^ The data reported in this paper suggest that by interfering with expression and likely redox state of PDI, GSH‐C4 can inhibit influenza A virus replication also in old mice.

Actually, in this paper we show that the inhibitory effect of GSH‐C4 treatment on influenza virus infection in old mice is more complex than merely inhibiting viral replication; in fact, it influenced the host immune response by increasing both IgA in BALF as well as IgG1 and IgG2a in plasma. This aspect is important since antibodies possess neutralizing properties, but IgG2a can stimulate antibody‐dependent cell‐mediated cytotoxicity and opsonophagocytosis by macrophages, contributing to the clearance of influenza virus from infected hosts.^26^, ^27^ Moreover, stimulation of IgG2a antibodies has been associated with increased efficacy of influenza vaccination^27^; for this reason, the finding that GSH‐C4 treatment can increase both IgG subtypes, and in particular IgG2a, deserves further studies to investigate the use of GSH‐C4 to enhance influenza vaccine‐specific IgG responses particularly in the elderly.

In addition, IgA induction in the mucosal tissue by GSH‐C4 treatment is another important point to consider given the fact that the main entrance for many pathogens such as influenza is the mucosal tissues. IgA can neutralize influenza viruses at the mucosal interface, even before they actually enter the host by crossing the mucosal barrier, thus existing IgA is the first line of defense upon reinfection or infection after vaccination. Moreover, IgA can effectively clear the virus in infected epithelial cells.^28^

Furthermore, GSH‐C4 treatment can influence the production of Th1/Th2 cytokines. The increase in IL‐2 concentration is particularly interesting considering that the production of this cytokine declines with age and that it is lower in old mice infected with influenza virus*.* 29 Moreover, we establish a link between the increased GSH content in the lungs and elevated production of IL‐12 in the BALF of influenza virus infected old mice treated with GSH‐C4. Hence, in agreement with previous works showing that GSH concentration influence IL‐12 production and Th response patterns,^4^, ^7^, ^8^, ^9^ we can conclude that in old mice infected with influenza virus, Th1/Th2 cytokine production can be also influenced by the redox state. In summary, GSH‐C4 treatment, by enhancing intracellular GSH content, elicited strong IgA and cellular immune responses that resulted in reduced lung virus titers in old mice (Table 2).

**Table 2:** Effect of GSH‐C4 treatment on old mice infected with PR8

	GSH‐C4 treatment
GSH concentration in lymph nodes and lungs	>
Th1/Th2 ratio in BALF	>
IL‐2 and IL‐12 pg/viral copies in BALF	>
IgA titer/viral copies in BALF	>
IgG1 and IgG2a in plasma	>
Viral M1 RNA copies in lungs	<
Body weight and body temperature loss	<

In our study, the senescent mouse model allowed us to obtain important insight to knowledge of the role of GSH in the immune defenses to invading pathogens. The murine model has been widely employed as a model for the investigation of aging, as well as immune response to influenza virus infection.^30^, ^31^ Recently, comparative studies on the immune responses to IAV infection between aged and young mice highlighted the relevance of mice as model for human aging and outcomes to IAV infection.^33^, ^34^

So, as widely reported,^4^, ^7^, ^8^, ^9^, ^15^ the results described in this paper, reinforce the conclusion that GSH exerts an important role in antiviral immunity, not simply as ROS scavenger, but by influencing the antiviral GSH‐dependent signaling pathways impaired by the virus. Therefore, the use of a molecule as GSH‐C4, equipped with both antiviral and immunomodulatory activity, may constitute a goal in the fighting influenza virus infection in aged people. The application of GSH‐C4 as vaccine adjuvant/immunomodulator in the prevention and treatment of influenza infection could be further explored in particular in elderly people where an unbalanced Th1/Th2 immune response is one of the reasons for the decline in function of the immune system leading to the failure of the vaccines commonly used.

## CONFLICT OF INTEREST

The author declare that they have no conflict of interest.

## AUTHOR CONTRIBUTIONS

L. Nencioni, A. Fraternale designed research; L. Nencioni, A. Fraternale, P. Checconi, and S. Brundu analyzed data; D. Amatore, I. Celestino, S. Brundu, L. Galluzzi, P. Coluccio performed research; L. Nencioni, A. Fraternale, A.T Palamara, and M. Magnani wrote the paper; L. Nencioni, A. Fraternale, D. Amatore, and I. Celestino contributed equally to the work.
